# Methotrexate‐associated lymphoproliferative disorder: A rare pancreatic tumor diagnosed via endoscopic ultrasound‐guided fine‐needle biopsy

**DOI:** 10.1002/deo2.346

**Published:** 2024-03-05

**Authors:** Koh Kitagawa, Masafumi Oyama, Shohei Asada, Shinya Sato, Tatsuya Nakatani, Naoki Nishimura, Yuka Mui, Jun‐ichi Hanatani, Maiko Takeda, Hitoshi Yoshiji

**Affiliations:** ^1^ Department of Gastroenterology Nara Medical University Nara Japan; ^2^ Department of Diagnostic Pathology Nara Medical University Nara Japan

**Keywords:** biopsy, Epstein–Barr virus infection, EUS‐guided tissue acquisition, methotrexate‐associated lymphoproliferative disorder, pancreatic neoplasms

## Abstract

A 73‐year‐old woman with a history of rheumatoid arthritis treated with methotrexate (MTX) for the last 10 years was referred to our hospital for a pancreatic tumor examination. Contrast‐enhanced abdominal computed tomography revealed a 20‐mm‐diameter hypovascular tumor in the pancreatic tail. A hypoechoic mass with heterogeneous internal echo was found on an endoscopic ultrasound (EUS). An EUS‐guided fine‐needle biopsy (EUS‐FNB) was performed with a 22‐gauge Franseen‐tip needle. Histologic examination of EUS‐FNB specimens from the pancreatic tumor revealed the proliferation of atypical spindle cells. Immunohistochemical staining for CD20 and Ki‐67 was positive in the atypical cells. Immunohistochemical staining for CD3 was partially positive in the atypical cells. Epstein–Barr virus‐encoded RNA in situ hybridization showed positive staining. MTX‐related lymphoproliferative disorder (MTX‐LPD) with Epstein‐Barr virus infection was diagnosed. MTX treatment was immediately discontinued, and treatment was initiated by a hematologist. However, her condition rapidly deteriorated, and she died of multiple organ failure 4 weeks after diagnosis. MTX‐LPD can complicate gastrointestinal lesions. However, most lesions are localized in the stomach and rarely complicate pancreatic lesions. MTX‐LPD is classified as an “iatrogenic” LPD. Therefore, immediate action, such as MTX discontinuation, is necessary. In conclusion, endoscopists should be aware that MTX‐LPD lesions can occur in the pancreas and gastrointestinal tract. Moreover, EUS‐FNB can be useful in the diagnosis of this rare pancreatic tumor.

## INTRODUCTION

Methotrexate (MTX) is an anticancer drug classified as a folate metabolism antagonist developed approximately 70 years ago. It is now widely used as a first‐line treatment for patients with rheumatoid arthritis (RA) having a poor prognosis. However, this drug has been associated with several adverse events, such as severe myelosuppression, MTX pneumonia, and infectious pneumonia. Furthermore, one study showed that patients receiving MTX can develop MTX‐associated lymphoproliferative disorder (MTX‐LPD),^1^ which can complicate gastrointestinal lesions^2^ but rarely pancreatic lesions. Herein, we report a case of MTX‐LPD complicated by a rare pancreatic lesion.

## CASE REPORT

**FIGURE 1:**
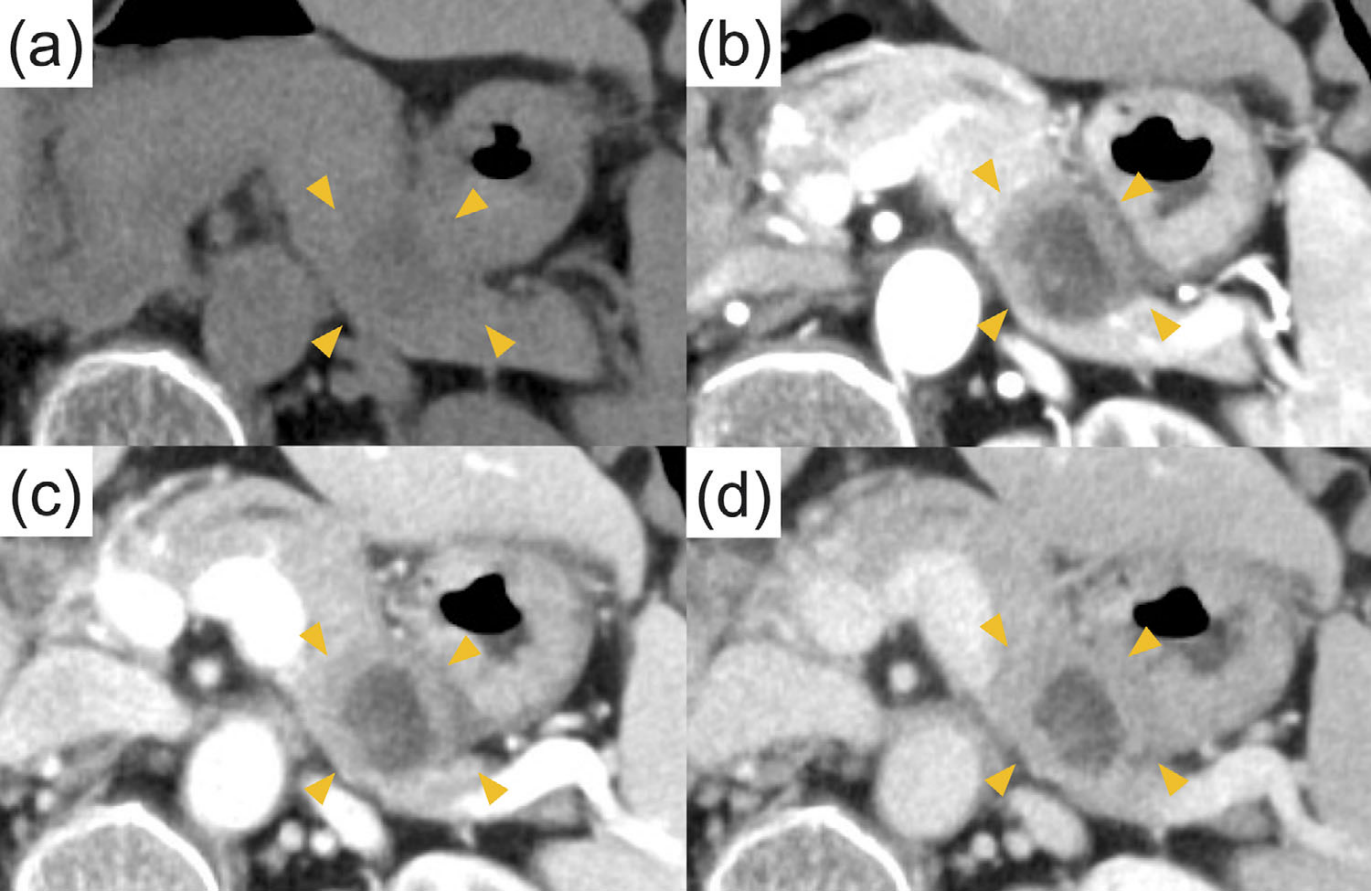
Abdominal computed tomography results: (a) noncontrast‐enhanced image; (b) late arterial phase; (c) portal phase; (d) delayed phase. A 20‐mm‐diameter hypovascular tumor is seen in the pancreatic tail (orange arrowheads). The lesion was round in shape, and a low‐density area suspected of necrosis or fluid retention was observed inside the lesion.

**FIGURE 2:**
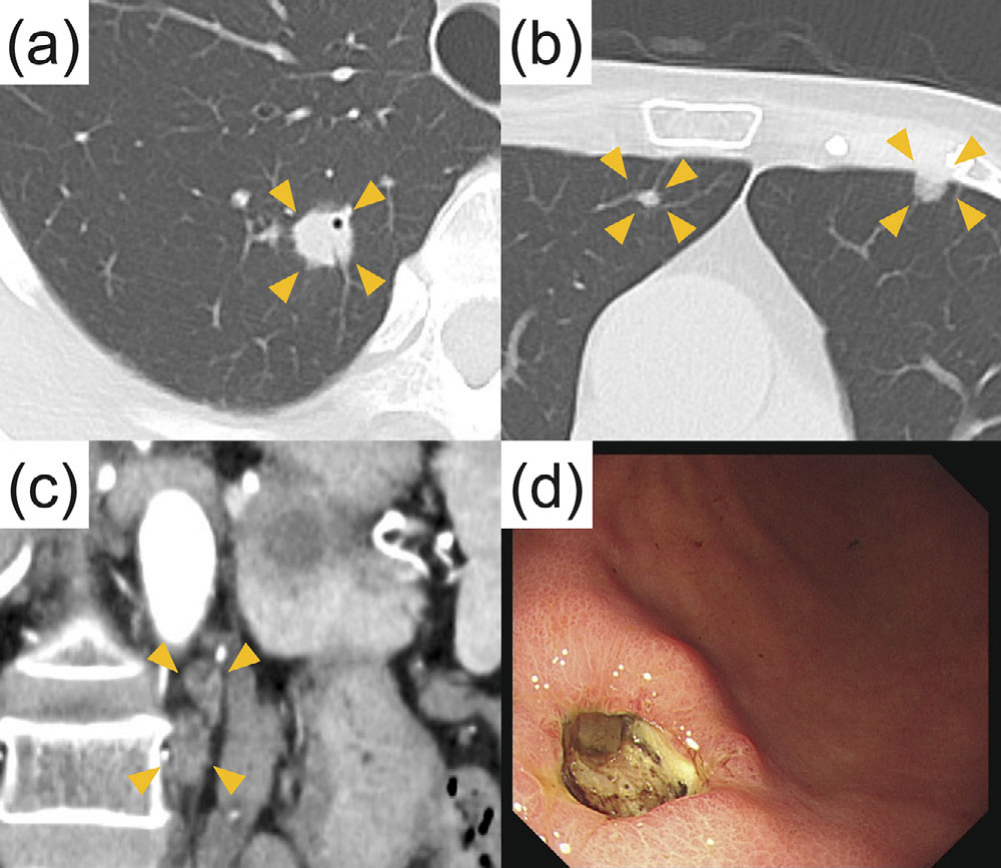
(a, b) Chest computed tomography results: Multiple round masses were found in both lungs (orange arrowheads), raising suspicions of metastatic lung cancer. (c) Abdominal computed tomography results (late arterial phase). Multiple enlarged lymph nodes were found around the aorta (orange arrowheads). (d) Images obtained following upper gastrointestinal endoscopy performed concurrently with endoscopic ultrasound. A solitary ulcer on the anterior wall of the greater curvature in the lower gastric body was detected.

**FIGURE 3:**
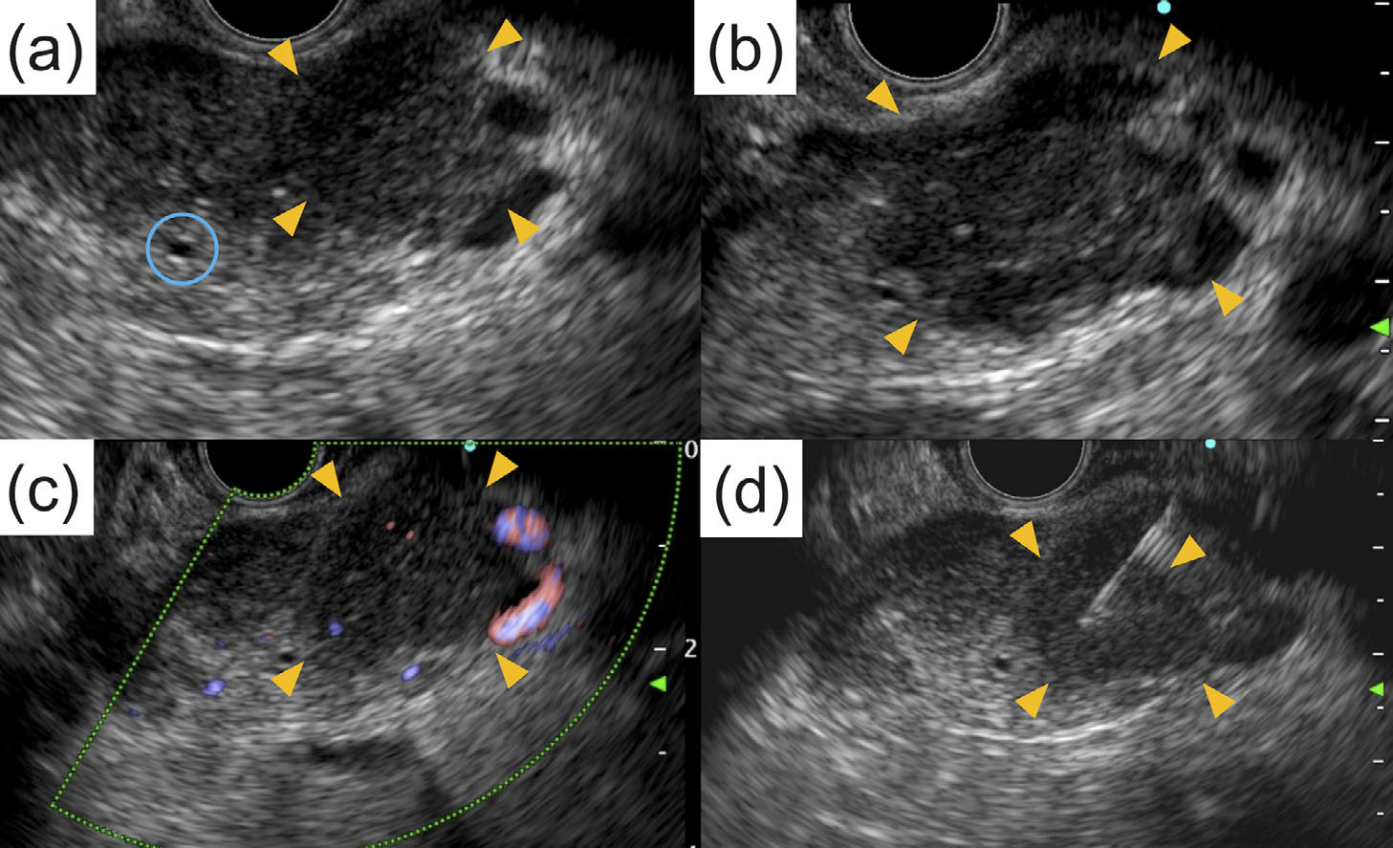
(a, b) Endoscopic ultrasound image (tissue harmonic echo imaging, penetration mode). A well‐defined hypoechoic mass was observed in the pancreatic tail (orange arrowheads). The tumor margins had an irregular morphology and were clearly demarcated from the surrounding normal pancreatic parenchyma. Internal echoes were heterogeneous. The main pancreatic duct was not involved in the lesion and was not dilated (blue circle). Lesion invasion into the gastric wall was not found. (c) H‐FLOW mode. Tumor blood flow was not extremely high. (d) An endoscopic ultrasound‐guided fine‐needle biopsy was performed.

**FIGURE 4:**
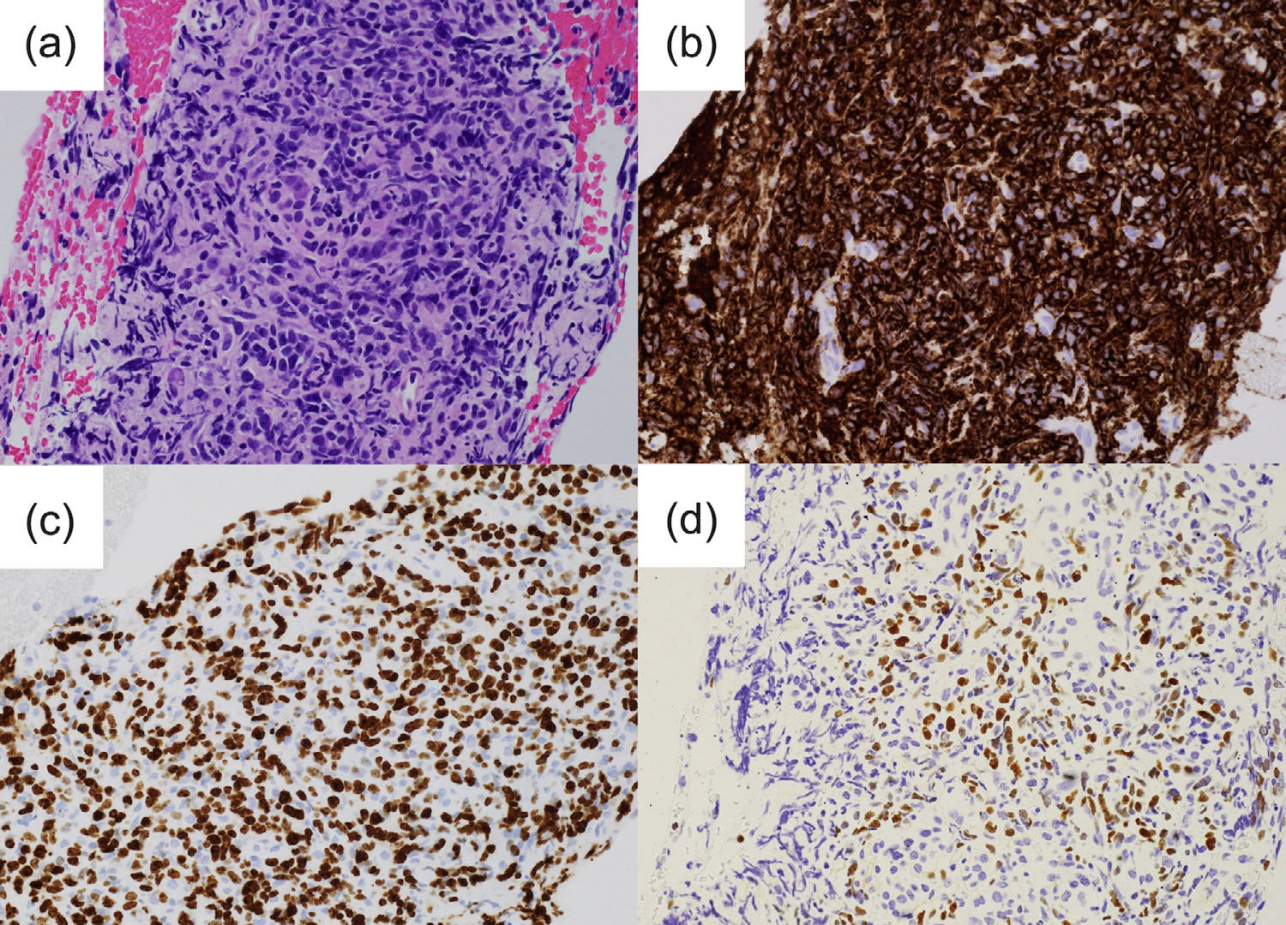
Histologic examination of biopsy specimens from the pancreatic tumor. (a) Hematoxylin–eosin staining showing spindle‐shaped atypical cell proliferation (magnification, 40×). (b) Immunohistochemical (IHC) staining for CD20 is positive in the atypical cells (40×). (c) Immunohistochemical staining for Ki‐67 is positive in the atypical cells (40×). (d) Epstein–Barr virus‐encoded RNA in situ hybridization showing positive staining (40×).

A 73‐year‐old woman with a history of RA treated with oral MTX (8 mg/week) and prednisolone (5 mg/day) within the last 10 years was referred to our hospital after a pancreatic tumor was incidentally detected on a screening abdominal ultrasound. There were no lymph nodes palpable from the body surface. Skin rashes or skin ulcers were not observed. The laboratory findings upon admission were as follows: white blood cell count, 3,800/μL; hemoglobin level, 10.4 g/dL; platelet count, 197,000/μL; lactate dehydrogenase, 437 U/L; C‐reactive protein level, 0.78 mg/dL; carcinoembryonic antigen, 2.7 ng/mL; carbohydrate antigen 19‐9, 71 U/mL; immunoglobulin (Ig) G4, 17.2 mg/dL; and soluble interleukin‐2 receptor, 3548 U/mL. The serum Epstein–Barr nuclear antigen‐antibody test and viral capsid antigen‐IgG antibody test had positive results. Contrast‐enhanced abdominal computed tomography (CT) revealed a 20‐mm‐diameter hypovascular tumor in the pancreatic tail (Figures 1a–d). The lesion was round in shape with a low‐density area suspected of necrosis or fluid retention inside the lesion. On the caudal side of the pancreas, no parenchymal atrophy or ductal dilatation was observed. However, multiple round masses were observed in both lungs (Figures 2a,b). In addition, multiple enlarged lymph nodes were found around the aorta (Figure 2c). Based on our examinations, we suspected the patient to have a special type of pancreatic cancer, such as neuroendocrine carcinoma or acinar cell carcinoma of the pancreas with multiple lung and lymph node metastases, and decided to perform endoscopic ultrasound‐guided fine‐needle biopsy (EUS‐FNB). Upper gastrointestinal endoscopy simultaneously performed with EUS‐FNB revealed a solitary ulcer on the anterior wall of the greater curvature in the lower gastric body (Figure 2d). EUS was performed using an echoendoscope (GF‐UCT260; Olympus Medical) connected to an ultrasound scanner (EU‐ME2; Olympus Medical; Figure 3). A well‐defined hypoechoic mass was observed in the pancreatic tail. Internal echoes were heterogeneous. The main pancreatic duct was not involved in the lesion and was not dilated. In the H‐FLOW mode, the tumor blood flow was not extremely high. Then, EUS‐FNB was performed using a 22‐gauge Franseen‐tip needle (Acquire; Boston Scientific Corporation). Histologic examination of EUS‐FNB specimens from the pancreatic tumor revealed the proliferation of atypical spindle cells (Figure 4a). Furthermore, atypical cells tested positive for CD20 and Ki‐67 (Figure 4b,c) and partially positive for CD3 (Figure S1a) on immunohistochemical (IHC) staining. However, the same cells tested negative for cytokeratin AE1/AE3 on IHC staining (Figure S1b). In addition, in situ hybridization showed positive staining for Epstein–Barr virus (EBV)‐encoded RNA (Figure 4d). Thus, a diagnosis of polymorphic MTX‐LPD with EBV infection was established. Consequently, MTX treatment was immediately discontinued, and treatment was initiated by a hematologist. Given the rapid deterioration of her general condition, our patient could not receive potent chemotherapy and was admitted to the intensive care unit for multidisciplinary treatment, including steroid pulse therapy. However, her condition rapidly deteriorated, and she died of multiple organ failure 4 weeks after diagnosis.

## DISCUSSION

MTX‐LPD frequently involves the extranodal organs throughout the body. Among the extranodal occurrences of MTX‐LPD, pulmonary involvement is most frequently observed.^1^, ^3^ MTX‐LPD can indeed complicate gastrointestinal lesions.^4^ These lesions are commonly localized in the stomach^2^ and can rarely complicate pancreatic lesions. In our case, the patient also had a concurrent gastric ulcer, with a biopsy from the same site yielding the same results as the pancreatic lesion. Therefore, the gastric lesion was also diagnosed as MTX‐LPD.

MTX‐LPD has been classified as an “iatrogenic” LPD.^5^ Therefore, immediate action, such as MTX discontinuation, is necessary. MTX‐LPD often enters remission after MTX is discontinued.^1^ Although the cause of LPD still remains unclear, studies have indicated that it can be caused by immune abnormalities associated with RA, complications of EBV infection, and the immunosuppressive effects of therapeutic drugs, such as MTX.^1^ MTX‐LPD is classified into several types based on histological characteristics, with diffuse large B‐cell lymphoma (DLBCL) as the most common type.^1^ Kurita et al. showed that the histological classification of MTX‐LPD is important and that the prognosis after MTX‐LPD discontinuation in reactive lymphoid hyperplasia is better than that in DLBDL.^1^ They also reported that the international prognostic indicator risk for DLBCL is a predictive factor of MTX‐LPD progression after MTX withdrawal. In contrast, they showed that age above 70 years and extensive necrosis for poly‐LPD are predictive factors for MTX‐LPD progression. Furthermore, Yamakawa et al. revealed that patients with an EBV‐positive mucocutaneous ulcer had better clinical outcomes than other patients with MTX‐LPD when MTX was withdrawn.^6^ However, it is not easy to accurately predict the prognosis. If there is no improvement, lesion biopsy should be performed again or chemotherapy must be administered. In our case, the obtained FNB specimen did not lead to the diagnosis of DLBCL but to poly‐LPD, and there was no necrosis in the specimen. Therefore, it was challenging to predict rapid progression in our case. It is also possible that the patient's condition rapidly transformed to DLBCL after EUS‐FNB. No evidence supports the performance of periodic imaging studies for the early detection of MTX‐LPD development in patients taking MTX. However, LPD occurrence should be suspected if unexplained fever, night sweats, or enlarged lymph nodes are observed in patients with MTX administration. Moreover, abnormal leukocyte fractions, anemia, and elevated LDH on blood tests should also be a cause for suspicion. Should such signs be present, imaging studies should be promptly performed.

Recently, the development of various EUS‐FNB needles has considerably improved the diagnostic rates of pancreatic tumors.^7^ These novel FNB needles are geometrically designed to obtain larger quantities of higher‐quality core samples. Therefore, previous reports have shown that the use of the FNB needles improves the accuracy of diagnosing lymphadenopathies, which were difficult to diagnose with conventional fine‐needle aspiration needles.^8^ Clinicians should also confirm the prolonged usage of MTX by the patients. If a tumor is found in a patient with prolonged usage of MTX, a sufficient tissue sample should be obtained and the necessary IHC should be performed, considering the possibility of MTX‐LPD.

MTX‐LPD of the pancreas is exceedingly rare, with no reports to date having presented detailed imaging findings of MTX‐LPD. In our case, the tumor was observed as a hypovascular round lesion on contrast‐enhanced CT. We observed an internal low‐density area and a “bull's‐eye” appearance, but it was impossible to determine whether these were common findings in pancreatic MTX‐LPD based on the examination results of this case alone. However, EUS revealed a hypoechoic tumor with a heterogeneous internal echo. However, an internal low‐density area was observed on the CT image. The reason for this discrepancy between CT and EUS findings remains unknown because a surgical specimen was not obtained. Abdominal magnetic resonance imaging might have been useful for detailed imaging. However, it could not be performed considering the rapid deterioration of the patient's general condition.

In conclusion, endoscopists should be aware that MTX‐LPD lesions can occur in the pancreas and gastrointestinal tract. Moreover, EUS‐FNB can be useful in diagnosing this rare pancreatic tumor.

## CONFLICT OF INTEREST STATEMENT

None.

## Supporting information


**Figure S1** Histologic examination of biopsy specimens from the pancreatic tumor. (a) Immunohistochemical (IHC) staining for CD3 is partially positive in the atypical cells (40×). (b) IHC staining for cytokeratin AE1/AE3 is negative (40×).
