# Supplementary figures and images for: Methotrexate‐associated lymphoproliferative disorder: A rare pancreatic tumor diagnosed via endoscopic ultrasound‐guided fine‐needle biopsy
Source: DEN Open. 2024 Mar 5;4(1):e346. doi: 10.1002/deo2.346 (PMC10914123; doi:10.1002/deo2.346)

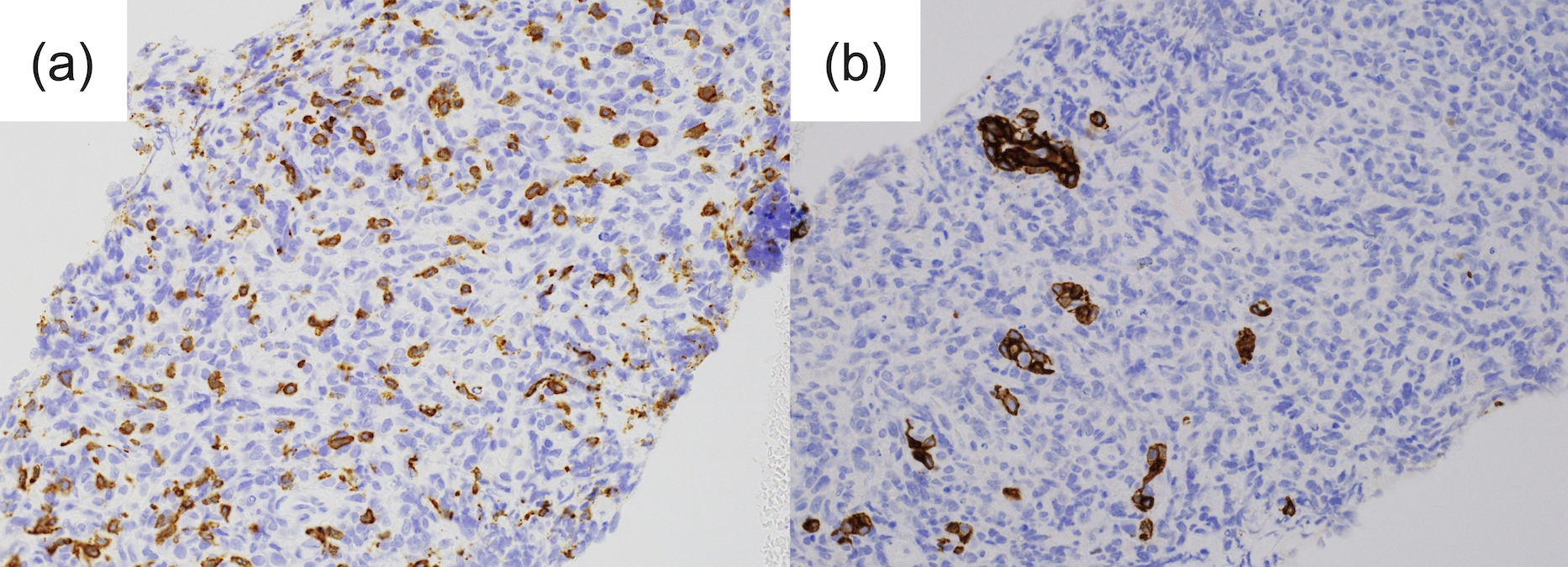

Supplement: Supplementary file 1 — Figure S1 Histologic examination of biopsy specimens from the pancreatic tumor. (a) Immunohistochemical (IHC) staining for CD3 is partially positive in the atypical cells (40×). (b) IHC staining for cytokeratin AE1/AE3 is negative (40×). [file DEO2-4-e346-s001.tiff]
